# The association between parenteral nutrition and pancreatic injury in adult patients: a retrospective observational study

**DOI:** 10.1186/s12986-022-00706-z

**Published:** 2022-10-31

**Authors:** Xiao-min Zhang, Yi-quan Zhou, Yan-ping Wan, Hao-jie Li, Zhi-qi Chen, An-qi Song, Mo-lian Tang, Renying Xu, Wei Cai

**Affiliations:** 1 Department of Pediatric Surgery, School of Medicine, Xin Hua Hospital, Shanghai Jiao Tong University, 200092 Shanghai, China; 2 Department of Clinical Nutrition, Ren Ji Hospital, School of Medicine, Shanghai Jiao Tong University, 200127 Shanghai, China; 3 Shanghai Key Laboratory of Pediatric Gastroenterology and Nutrition, Shanghai, China; 4 Shanghai Institute of Pediatric Research, No. 1665, Kong Jiang Road, 200092 Shanghai, China

**Keywords:** Parenteral nutrition (PN), Lipase, Pancreatic amylase, Total bile acids

## Abstract

**Background and objective:**

Patients on parenteral nutrition (PN) are at high risk of both liver and pancreatic injury. More efforts were focused on liver, however, limited data is available to evaluate the effects of PN on pancreas. Thus, we performed a retrospective observational study to evaluate the association between PN and pancreatic injury in Chinese adult patients.

**Methods:**

Adult patients (18–80 years), who received PN for a week or longer, and with repeated measurements of pancreatic enzymes, were included in the analysis. Pancreatic injury was confirmed by serum level of pancreatic amylase (P-AMYwas 53 U/L or higher) or lipase (LP was 63 U/L or higher), which were evaluated at baseline and following every week during PN duration. Age, sex, body weight, height, diagnosis of diseases, history of diseases, surgery, white blood cell, c-reactive protein, liver and renal function, fasting blood glucose, lipid profile, and daily energy supplied by PN and enteral nutrition were abstracted from medical records.

**Results:**

A total number of 190 adult patients (125 men, 65 women) were included in the study. The average age and BMI were 61.8 ± 13.0 years and 21.7±3.3 kg/m^2^, while medium serum level of P-AMY and LP were 29.0 U/L (quartile range: 18.0, 47.0) and 33.0 U/L (quartile range: 19.0, 58.0), respectively at baseline. The median duration of PN was 15 days (quartile range: 11.0, 21.0). The prevalence of pancreatic injury was 42.1% (80/190) while it was 28.4% (54/190), 43.3% (77/178), 47.8% (44/92) after one-, two-, and three-week or longer PN adminstration. The proportion of daily energy supplement by PN (OR = 3.77, 95%CI: 1.87, 7.61) and history of infection were positively (OR = 3.00, 95%CI: 1.23, 7.36), while disease history for diabetes mellitus (OR = 0.38, 95%CI: 0.15, 0.98) and cancer (OR = 0.46, 95%CI: 0.23, 0.95), were negetively associated with pancreatic injury. Total bile acids were associated with the increment of P-AMY (beta = 0.98, 95%CI: 0.39, 1.56) and LP (beta = 2.55, 95%CI: 0.98, 4.12) by multi-variate linear regression.

**Conclusion:**

PN was associated with pancreatic injury, as demonstrated by the increase of both serum P-AMY and LP.

**Supplementary Information:**

The online version contains supplementary material available at 10.1186/s12986-022-00706-z.

## Introduction

Parenteral nutrition (PN) is a crucial intervention for patients who cannot meet energy and nutrient requirements through oral intake or tube feeding. Cumulative evidences have proved that PN does improve nutritional status, shorten hospital stay, and decrease mortality [1–3]. However, inappropriate implementation of PN may also cause a series of complications, including PN-associated liver diseases, catheter-related bloodstream infections, and injury to the intestinal barrier [4, 5]. PN-associated adverse effects have been found to be caused mainly by the inhibition of digestive enzymes and biliary sludge, with numerous studies referring to hepatobiliary dysfunction and cholestasis [6–8]. Pancreas performs both endocrine and exocrine functions, providing digestive juices and bile acids from the bile duct to hydrolyze macromolecules [9]. Therefore, it is highly likely that PN might affect the exocrine pancreas as a consequence of bypassing normal digestion or cholestasis, which results in an impaired digestive and absorptive function.

Several studies have reported that PN suppressed the function of the exocrine pancreas. The first animal study was performed in 1977, evaluating the association between PN and dog pancreatic secretion and found that pancreatic secretion reduced after initiation of PN [10]. Niederau et al. ^11^conducted a self-control trial on healthy volunteers in 1985 and found that amino acids significantly stimulated the pancreatic secretion of trypsin and chymotrypsin, but without obvious effects of intravenous lipid or glucose on pancreatic secretion. Most published studies focused on the effects of intravenous single macronutrients, such as glucose, lipid, or amino acids on pancreatic exocrine function. However, all-in-one mixtures are recommended by ASPEN, ESPEN, and CSPEN to assure stable administration of macro- and micronutrients [11–13]. Data regarding the association between PN administrated by all-in-one mixtures and pancreas are limited. Therefore, we performed the current retrospective observational study to evaluate the association between PN and pancreatic exocrine function, which was regularly measured by P-AMY and LP [14–16], in a group of Chines adult patients on PN for one week or longer.

## Subjects and methods

### Study population

This is a single-center retrospective obeservational study. The study protocol was approved by the Ethics Committee of Ren Ji Hospital (Project no. KY2021-048) in accordance with the Declaration of Helsinki. As a retrospective data analysis, patients’ written consent was waived by the same committee .

All participants were recruited from Ren Ji Hospital from 1 to 2020 to 1 Dec 2021. The inclusion criteria were as following: (1) aged between 18 and 80 years; (2) who received PN for one week or longer (3) whose serum level of P-AMY and LP were repeatedly measured. The exclusion criteria were those: (1) aged less than 18 years or more than 90 years; (2) with missing data; (3) with estimated glomerular filtration rate (eGFR) < 30 ml/min/1.73m^2^, because urinary excretion of lipase decreased in patients with chronic kidney diseases [17]; (4) with serum level of alkaline phosphatase and gamma-glutamyl transferase ≥ 1.5 ULN (upper limit of normal value) and serum level of direct bilirubin ≥ 2 ULN based on the diagnostic criteria of cholestasis [7, 8]; (5) with history of confirmed liver, gall bladder and pancreas injuries (e.g., cancer, acute or chronic inflammation, trauma, abused drugs, autoimmune disease), rheumatic disease, organ transplant and pregnancy; (6) admitted to critical care units.

**Fig. 1:**
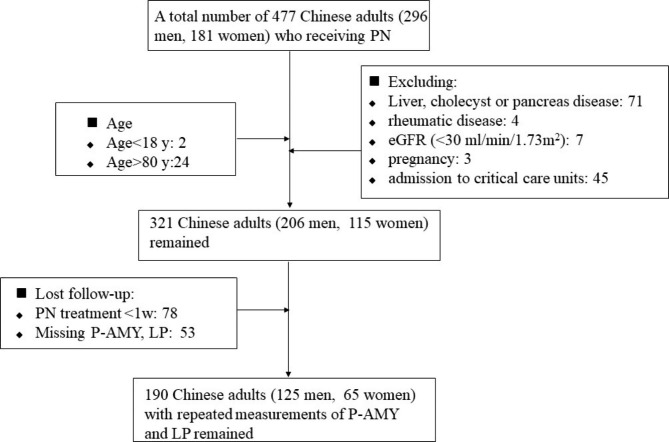
The process of sample recruitment. Abbreviation: eGFR, estimated glomerular filtration rate; P-AMY, pancreatic amylase; LP, lipase;

The intial inclusion resulted in 477 patients receiving PN, which was trimmed to 321 after sequential inclusion. After excluding those who lost follow-up, the final number of participants were 190. (Fig. 1). There were no significant differences among the participants in gender ratio, disease history and major clinical parameters in and out of the study. (Supplementary Table 1. The comparison of baseline characteristics between participants in and out of the study).

### PN regimens and follow-up

A professional nutrition support team reviewed the patients and prescribed PN regimens. The indication for PN was based on the insufficient oral intake or tube feeding that cannot meet 60% of the daily requirement [12]. These patients were reviewed daily by the same nutrition support team. Other sources of energy supply, such as enteral nutrition and oral intake, were also recorded and calculated. For each patient, the duration of PN, enteral nutrition and oral intake were accounted separately from the initial to the last day of PN treatment.

### Biochemical parameters

Biochemical parameters were monitored and recorded at baseline and were measured repeatedly for seven consecutive days after initiation of PN. Blood samples were drawn after at least an 8 h fast. Baseline parameters were analyzed within 24 h before PN (day 0). Subsequent analyses were performed at a 7-day interval until PN was stopped (e.g., day 7, day 14). Serum levels of P-AMY and LP were measured by the enzyme colorimetry analysis (Roche 701 Bioanalyzer, Roche, UK). Serum levels of alkaline phosphatase, gamma glutamyl-transferase, direct bilirubin, total bilirubin, bile acids, alanine transferase and aspartate transferase were measured by enzyme-linked immunosorbent assay (Roche 701 Bioanalyzer, Roche, UK). Fasting blood glucose, albumin, pre-albumin, and creatinine were also measured by enzyme-linked immunosorbent assay (Roche 701 Bioanalyzer, Roche, UK). The eGFR was calculated using the Chronic Kidney Disease Epidemiology Collaboration 2-level race Eq. 1 [8]. White blood cells were measured using an automated hematology analyzer (DxH 690T, Beckman Coulter, USA). The concentration of C-reaction protein was measured by the immunoturbidimetric method (CardioPhase hsCRP kit, Siemens Healthcare Diagnostics Products GmbH, Germany). All the measurements above were completed in the Clinical Laboratory of the Ren Ji Hospital.

### Other clinical information

Age, sex, diagnosis, history of disease and surgery, body weight and height were recorded from the medical record. Gastrointestinal dysfunction is defined as the presence of diarrhea, vomiting, abdominal distension, or intestinal obstruction [18]. The IBW (ideal body weight) was calculated as [current body height (cm) − 80) × 0.7] in men and [current body height (cm) − 70) × 0.6] in women [19].

### Definition of pancreatic injury

Pancreatic injury were confirmed by serum of P-AMY (≥53 U/L) or LP (≥63 U/L) based on the normal range recommended by the Clinical Laboratory of Ren Ji Hospital. Serum levels of P-AMY and LP were repeated measured every 7 day [14, 15].

### Statistical analysis

All statistical analyses were performed using IBM SPSS Statistics 23 software (IBM, Armonk, New York, United States). For the hypothesis testing, a two-sided test with a 5% significant level was used. Data with normal distribution were presented as mean±standard and the differences between the groups were tested by non-paired student tests. Non-normally distributed data were presented in median and quartile ranges, and the differences between groups were tested using the Chi-square method.

We performed univariate and multivariable binary logistic regression modeling to evaluate the association between baseline clinical features and pancreatic injury. Potential parameters were selected by Univariable regression (*p* < 0.2) and refered to relatively clinical trials [20–22]. Nive parameters (sex, age, the proportion of daily energy supplement by PN, surgery, diabetes mellitus, cancer, infection, gastrointestinal dysfuction, fistula) entered multivariate binary logistic regression model. Risk factors for PN-related liver dysfunction were evaluated by Spearman correlation and linear regression analysis. Two-step linear regression modeling was performed to evaluate the association between the serum level of liver function and pancreatic enzymes. Univariable regression was first carried out to identify eligible factors based on *p* value (*p* < 0.2). Using the regression results, four parameters (alanine transferase, aspartate transferase, total bile acids, gamma glutamyl-transferas) were included in the multivariate regression model.

## Results

A total of 190 patients (125 men and 65 women) were included in this study. The average age and BMI were 61.8 ± 13.0 years and 21.7±3.3 kg/m^2^, while medium serum level of P-AMY and LP were 29.0 U/L (quartile range: 18.0, 47.0 U/L) and 33.0 U/L (quartile range: 19.0, 58.0 U/L), respectively at baseline. In the current study, 71.1% of the participants were fasted, 68.4% of them were with cancer, and 77.9% started PN after surgery. Baseline characteristics of biochemical indicators showed no statistical differences between groups. (Table 1).

**Table 1:** Baseline characteristics in 190 patients receiving parenteral nutrition

Variables	Pancreatic injury		*p* value
	No (n = 110)	Yes (n = 80)	
Clinical features			
Age, y	62.0±13.1	61.5±12.9	0.82
Sex, woman, %	27.3% (30)	43.8% (35)	0.02
BMI, kg/m^2^	21.7±3.3	21.7±3.4	0.91
Fasted, yes, %	63.6% (70)	81.3% (65)	0.008
Surgery, yes, %	78.2% (86)	77.5% (62)	0.91
Diabetes mellitus, yes, %	20.0% (22)	12.5% (10)	0.17
Cancer, yes, %	74.5% (82)	60.0% (48)	0.03
Infection, yes, %	14.5% (16)	23.8% (19)	0.11
Gastrointestinal dysfunction, yes, %	48.2% (53)	50.0% (40)	0.80
Fistula, yes, %	12.7% (14)	13.8% (11)	0.84
PN solution			
Duration of PN, d*	14 (10.0, 20.0)	15 (12.0, 22.0)	0.53
Duration of EN, d*	4.0 (0.0, 10.0)	0.5 (0.0, 9.0)	0.18
Duration of oral-intake, d*	2.0 (0.0, 10.0)	3.0 (0.0, 7.0)	0.55
Avg PN-E_CBW_, kcal/kg/d	18.1 ± 5.6	19.7 ± 5.3	0.04
Avg PN-E_IBW_, kcal/kg/d	17.6 ± 3.9	19.3 ± 3.7	0.003
Avg PN-E/total-E, %	68.0 ± 19.1	77.4 ± 15.3	0.001
Biomedical parameters			
WBC, 10^9^/L*	8.0 (6.0, 12.0)	9.8 (6.9, 13.1)	0.05
CRP, mg/L*	39.1 (10.2, 101.3)	40.2 (13.4, 115.0)	0.95
FBG, mmol/L	6.2 ± 2.5	6.5 ± 2.9	0.46
ALT, IU/L*	15.0 (10.0, 23.3)	19.5 (9.3, 36.8)	0.51
AST, IU/L*	18.5 (13.0, 28.0)	21.5 (15.3, 37.8)	0.08
AKP, IU/L*	69.0 (52.0, 88.8)	68.0 (54.3, 85.8)	0.47
γ-GT, IU/L*	25.0 (14.0, 44.5)	22.5 (13.0, 62.0)	0.38
TBI, µmol/L*	12.3 (8.4, 16.0)	10.3 (7.6, 14.7)	0.96
DBI, µmol/L*	4.8 (3.2, 6.5)	4.4 (3.2, 7.4)	0.46
TBA, µmol/L*	2.1 (0.6, 5.2)	1.7 (0.7, 4.7)	0.82
ALB, g/L	33.1 ± 4.9	32.3 ± 6.2	0.36
Pre-ALB, mg/L	151.2 ± 77.5	136.8 ± 59.8	0.17
TC, mmol/L	3.6 ± 1.3	3.2 ± 0.9	0.03
TG, mmol/L*	1.2 (0.8, 1.6)	1.2 (0.9, 1.6)	0.32
HDL, mmol/L	0.9 ± 0.3	0.8 ± 0.3	0.02
LDL, mmol/L	2.0 ± 1.1	1.7 ± 0.7	0.04
FFA, mmol/L	0.6 ± 0.3	0.6 ± 0.3	0.80
eGFR, ml/min/1.73m^2^	108.8 ± 59.6	98.7 ± 48.9	0.22

Note:

1.* non-normal distribution, data is shown as median and quartile range

2. Abbreviation: BMI, body mass index; EN, enteral nutrition; WBC, white blood cell; CRP, C-reactive protein; ALT, alanine transferase; AST, aspartate transferase; AKP, alkaline phosphatase; γ-GT, gamma glutamyl-transferase; TBI, total bilirubin; DBI, direct bilirubin; TBA, total bile acids; ALB, albumin; Pre-ALB, pre-albumin; TC, total cholesterol; TG, triglycerides; HDL, high-density lipoprotein; LDL, low-density lipoprotein; FFA, fatty free acids; FBG, fast blood glucose; eGFR, estimated glomerular filtration rate; P-AMY, pancreatic amylase; LP, lipase;

3. total-E, total energy means the sum of daily energy supply of PN plus EN plus oral-intake

4. Avg PN-E_CBW_: daily average energy supply of PN in kcal per kg based on current body weight; Avg PN-E_IBW_: daily average energy supply of PN in kcal per kg based on ideal body weight

5. PN-E/total-E, PN energy/total energy means the proportion of daily energy supply of PN in the total energy

6. Pancreatic injury is defined as confirmed by either abnormal elevated serum level of P-AMY or LP based on the normal range

7. Gastrointestinal dysfunction is defined as the presence of diarrhea, vomiting, abdominal distension, or intestinal obstruction

The median duration of PN was 15 days (quartile range: 11.0, 21.0 d). Daily energy supplement was 18.7 ± 5.5 kcal/kg/d by PN based on current body weight, while it was 18.3 ± 4.0 kcal/kg/d based on ideal body weight. The details for macronutrients supplement based on current and ideal body weight were shown in Table 1.

**Fig. 2:**
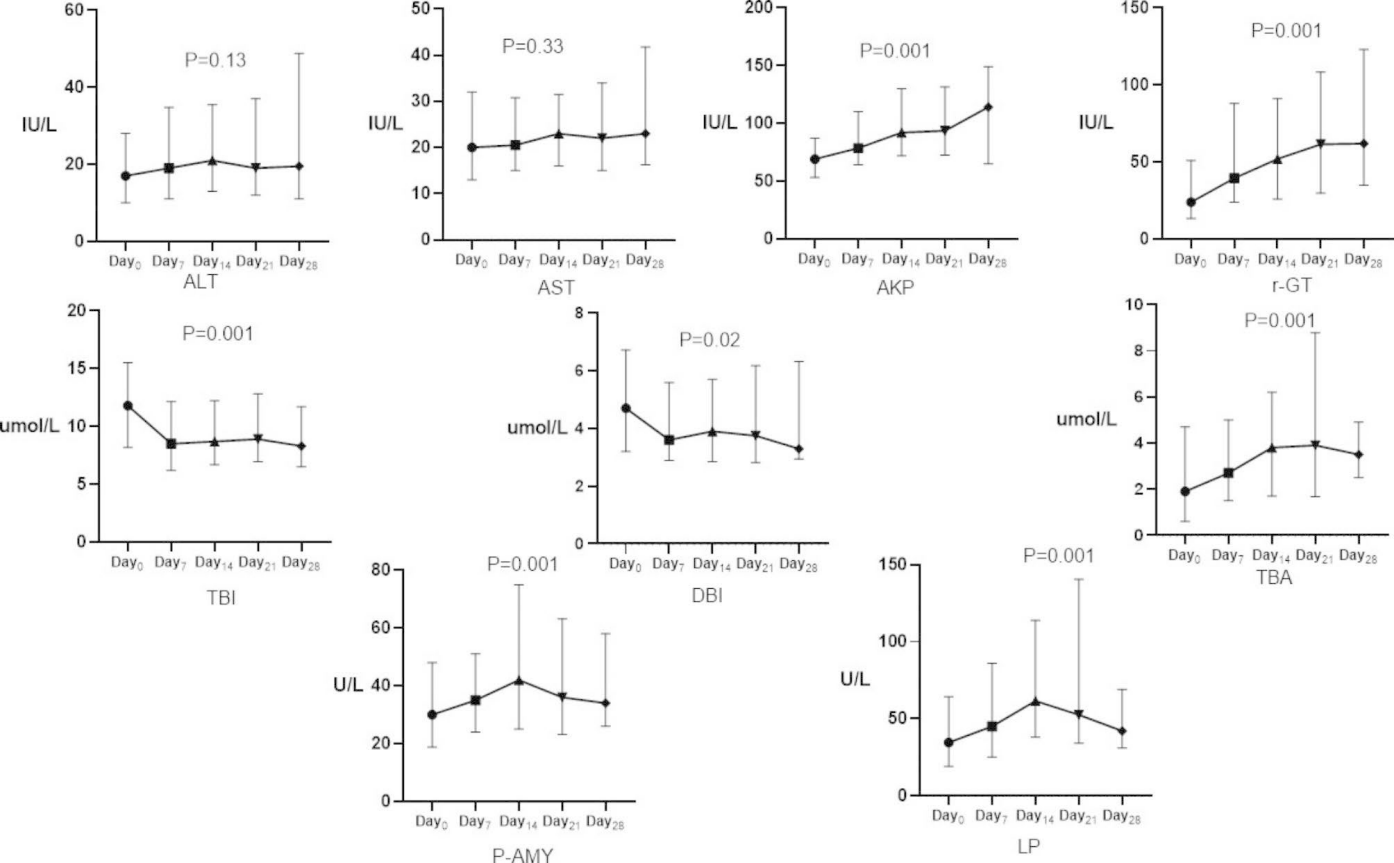
The curve of serum pancreatic enzymes and liver function during the PN treatment. Abbreviation: ALT, alanine transferase; AST, aspartate transferase; AKP, alkaline phosphatase; γ-GT, gamma glutamyl-transferase; TBI, total bilirubin; DBI, direct bilirubin; TBA, total bile acids; P-AMY, pancreatic amylase; P, lipase;

The prevalence of pancreatic injury was 28.4% (54/190), 43.2% (77/178), 47.8% (44/92) after one-, two-, three-week or longer administration of PN and the total prevalence was 42.1% (80/190). The proportion of daily energy supplement by PN (OR = 3.77, 95%CI: 1.87, 7.61) and history of infection were positively (OR = 3.00, 95%CI: 1.23, 7.36), while disease history for diabetes mellitus (OR = 0.38, 95%CI: 0.15, 0.98) and cancer (OR = 0.46, 95%CI: 0.23, 0.95), were negetively associated with pancreatic injury. Compared with men, women on PN were marginally associated with high risk of pancreatic injury (OR = 1.80, 95%CI: 0.92, 3.52) (Table 2). The increment of P-AMY and LP were associated with aspartate transferase (r=-0.39 and − 0.22, *p* < 0.05) and total bile acids (r = 0.22 and 0.25, *p* < 0.05) (Table 3). Serum levels of P-AMY, LP, gamma glutamyl-transferase, alkaline phosphatase, and total bile acids increased after PN administration, compared with baseline measurements (Fig. 2, all *p* < 0.05). Multivariate linear regression analysis showed that total bile acids were associated with the increment of P-AMY (β = 0.98, 95%C.I.:0.39 ~ 1.56) and LP (β = 2.55, 95%C.I.:0.98 ~ 4.12) (Table 4).

**Table 2:** The association between clinical parameters and pancreatic injury in 190 patients receiving parenteral nutrition: multivariate logistic regression

Variables			Groups	*p* value
Sex		men	1.0 (ref)	NA
		women	1.80 (0.92, 3.52)	0.08
Age, y		< 70	1.0 (ref)	NA
		≥ 70	0.77 (0.39, 1.55)	0.47
Avg PN-E/total-E, %		< 70	1.0 (ref)	NA
		≥ 70	3.77 (1.87, 7.61)	0.01
Surgery		no	1.0 (ref)	NA
		yes	2.05 (0.85, 4.91)	0.11
Diabetes mellitus		no	1.0 (ref)	NA
		yes	0.38 (0.15, 0.98)	0.04
Cancer		no	1.0 (ref)	NA
		yes	0.46 (0.23, 0.95)	0.04
Infection		no	1.0 (ref)	NA
		yes	3.00 (1.23, 7.36)	0.02
Gastrointestinal dysfunction		no	1.0 (ref)	NA
		yes	0.95 (0.47, 1.91)	0.88
Fistula		no	1.0 (ref)	NA
		yes	0.90 (0.33, 2.45)	0.83

Note:

1. NA, not applicable

2. PN-E/total-E, PN energy/total energy means the proportion of daily energy supply of PN in the total energy

>

**Table 3:** The correlation between the changes of P-AMY and LP with liver function in 190 patients receiving parenteral nutrition: univariate correlation analysis

Variables	ΔP-AMY		ΔLP	
	r	*p* value	r	*p* value
ΔALT	-0.11	0.14	0.04	0.55
ΔAST	-0.39	0.001	-0.22	0.002
ΔAKP	-0.04	0.59	0.03	0.73
Δγ-GT	0.03	0.71	0.14	0.06
ΔTBI	-0.03	0.71	-0.01	0.96
ΔDBI	0.02	0.82	0.04	0.62
ΔTBA	0.22	0.002	0.25	0.001

Note:

1. The changes of measurements were calculated by repeated value minus baseline value

2. Abbreviation: ALT, alanine transferase; AST, aspartate transferase; AKP, alkaline phosphatase; γ-GT, gamma glutamyl-transferase; TBI, total bilirubin; DBI, direct bilirubin; TBA, total bile acids; P-AMY, pancreatic amylase; LP, lipase;

**Table 4:** The association between the changes of P-AMY and LP with liver function in 190 patients receiving parenteral nutrition: linear regression analysis

		R	R^2^	F	*p* value	β	t	*p* value	95% C.I.
ΔP-AMY	Constant	0.53	0.28	17.15	0.001	4.86	1.74	0.08	-0.66 ~ 10.37
	ΔTBA					0.98	3.23	0.001	0.39 ~ 1.56
	ΔALT					0.42	3.54	0.001	0.19 ~ 0.65
	ΔAST					-0.75	-7.30	0.001	-0.95~-0.55
	Δγ-GT					0.02	0.52	0.60	-0.08 ~ 0.12
ΔLP	Constant	0.44	0.19	10.72	0.001	14.88	1.99	0.05	0.12 ~ 29.63
	ΔTBA					2.55	3.21	0.002	0.98 ~ 4.12
	ΔALT					1.01	3.19	0.002	0.39 ~ 1.63
	ΔAST					-1.42	-5.18	0.001	-1.96~-0.88
	Δγ-GT					0.19	1.56	0.12	-0.05 ~ 0.44

Note:

1. The changes of measurements were calculated by repeated value minus baseline value

2. Abbreviation: ALT, alanine transferase; AST, aspartate transferase; γ-GT, gamma glutamyl-transferase; TBA, total bile acids; P-AMY, pancreatic amylase; LP, lipase;

## Discussion

In this retrospective study of 190 Chinese adults patients receiving PN, we found that 42.1% (80/190) of participants were confirmed with pancreatic injury, as demonstrated by elevated serum levels of P-AMY or LP. Our results supported that PN was at least partially accountable for pancreatic injury.

Consistent with our results, a retrospective study (n = 102) reported that the prevalence of elevated serum levels of lipase and amylase was higher in patients with PN compared to those without PN [23]. Pancreatic enzymes were even higher as the diseases deteriorated 16]. Previou studies have confirmed that PN suppress the function of the exocrine pancreas [10, 24]. Although PN were thought to have no obvious effects on pancreatic secretion in humans [21, 22], our study demonstrated that long-term (≥ one week or longer) PN might lead to pancreatic injury. A series of studies suggested PN treatment could lead to dysfunction and atrophy of the pancreas. Fan et al. reported that pancreas weight and the concentration of pancreatic amylase were lower in the PN group compared with control group [14] and found that pancreatic atrophy and dysfunction might be the possible mechanism [25]. They concluded that intravenous hypertonic glucose resulted in atrophy of the pancreas by the suppression of the stimulatory effect of cholecystokinin on pancreas [26]. Gabrielson et al. found diffuse, moderate pancreatic epithelial cell necrosis, along with acinar atrophy and interstitial fibrosis in piglets underwent 3-week total PN [27]. Another animal study also confirmed pancreatic injury in rats [14] after 7-day total PN. Therefore, pancreatic functions as well as the effects on digestive enzymes and hormones, should be considered when administering PN.

Our study showed that the changes in serum P-AMY and LP were closely associated with the changes in the serum level of liver enzymes and total bile acids, which implied that PN-related elevation of pancreatic enzymes were linked with hepatic complications. This finding was supported by some previous studies. O’Keefe SJ et al. recruited 27 healthy men to evaluate pancreaticobiliary secretory and metabolic responses to enteral and parenteral feeding [28]. They found that intravenous feeding suppressed the secretion of both pancreatic enzymes and bile acid and lowered the plasma concentrations of gastrin and cholecystokinin. Meessen ECE et al. evaluated the effects of intravenous nutrition on circulating level of bile acids and gut hormones in 8 healthy lean men [29]. They found significant increases in bile acids after PN initiation. Bile acids could have harmful effects on pancreatic duct cells [30, 31]. Consistent with above-mentioned studies, we also observed that serum bile acids were significantly associated with serum level of pancreatic enzymes, which indicated that bile acids might play an important role in PN related pancreatic injury. Philip D Hardt et al. reviewed the studies on intensive care patients and draw a conclusion that elevation in enzyme levels could reflect pancreatic injury and the development of biliary sluge could be one of the contributing factors [32].

The strengthen of our study included its cohort study design and deliberated control of potential confoundings. However, some limitations must be concerned. First, as a retrospective study, 131 patients were exclude, which might cause bias. However, baseline characteristics between the patients in and out of the study were similar. Second, although hepato-pancreatic diseases and patients in critical care unit were excluded, patients included in the study tended to have more severe disease and had high risk of pancreatic injury. Third, we could not exclude the possibility that pancreatic injury was caused or was partially induced by infection. We collected data on inflammation biomarkers (e.g., white blood cell and C-reactive protein) and found no significant differences between the two groups (i.e., with and without pancreatic injury). A well-designed prospective study with randomized control to Total PN and other nutritional support is recommended to validate the findings of this study.

## Conclusion

PN could impair pancreatic exocrine function and pancreatic injury was usually accompanied with liver dysfunction. It is necessary to monitor serum level of pancreatic enzymes in order to early confirmation and treatment of pancreatic injury.

## Electronic supplementary material

Below is the link to the electronic supplementary material.

Supplementary Material 1. The comparison of baseline characteristics between participants in and out of the study

## Data Availability

The data and SAS code were available upon reasonable request (Renying Xu, email address: 721,001,735@shsmu.edu.cn).
