# Supplementary material for: The association between parenteral nutrition and pancreatic injury in adult patients: a retrospective observational study
Source: Nutr Metab (Lond). 2022 Oct 31;19:73. doi: 10.1186/s12986-022-00706-z (PMC9624056; doi:10.1186/s12986-022-00706-z)
Supplement: Supplementary file 1 — Supplementary Material 1. The comparison of baseline characteristics between participants in and out of the study [file 12986_2022_706_MOESM1_ESM.docx]

**Supplementary Table 1.** The comparison of baseline characteristics between participants in and out of the study

| Variables | Participants | | *p* value |
| --- | --- | --- | --- |
|  | in (n=190) | lost (n=131) |  |
| Age, y | 61.8±13.0 | 57.7±13.0 | 0.01 |
| Sex, woman, % | 34.2% (65) | 38.2% (50) | 0.42 |
| BMI, kg/m^2^ | 21.7±3.3 | 21.8±3.9 | 0.84 |
| Surgery, yes, % | 77.9% (148) | 71.8% (94) | 0.29 |
| Infection, yes, % | 18.4% (35) | 12.2% (16) | 0.15 |
| WBC, 10^9^/L* | 8.9 (6.2, 12.5) | 8.8 (6.6, 12.1) | 0.30 |
| ALT, IU/L* | 17.0 (10.0, 28.0) | 16.0 (10.0, 27.0) | 0.24 |
| AST, IU/L* | 20.0 (13.0, 32.0) | 18.0 (14.0, 32.0) | 0.55 |
| r-GT, IU/L* | 24.0 (13.0, 52) | 28.0 (18.0, 48.0) | 0.23 |
| TBI, μmol/L* | 11.9 (8.2, 15.5) | 9.5 (6.4, 14.0) | 0.15 |
| DBI, μmol/L* | 4.7 (3.2, 6.7) | 4.0 (2.9, 6.0) | 0.34 |
| TBA, μmol/L* | 1.9 (0.6, 4.7) | 1.5 (0.5, 3.9) | 0.19 |

**Note:**

1. * non-normal distribution, data is shown as median and quartile range

2. Abbreviation: **BMI**, body mass index; **WBC**, white blood cell; **ALT**, alanine transferase; **AST**, aspartate transferase; **γ-GT**, gamma glutamyl-transferase; **TBI,** total bilirubin; **DBI**, direct bilirubin; **TBA**, total bile acids
